# The Devitalization of the Dental Pulp

**Published:** 1892-08

**Authors:** W. D. Miller

**Affiliations:** BERLIN, GERMANY.


ARTICLE VI.

### THE DEVITALIZATION OF THE DENTAL PULP.

BY W. D. MILLER, M. D., PH. D., BERLIN, GERMANY.

The devitalization of the dental pulp has repeatedly been made the subject of communications to the dental journals, and of discussions in dental societies. Expression has often been given to the general complaint that application of arsenic to the dental pulp is too frequently followed by severe pain, and various means have been suggested for rendering that operation less trying to the patient.

My experience in the devitalization of pulps differs somewhat from that of many writers on the subject, in that I am able to perform the operation without pain; at least I have not had a single case in the last two years in which the patient has complained of severe pain after the application of arsenic, and in nearly all cases they have said they had felt nothing whatever. A description of the manner in which I devitalize pulps may, therefore, be of service to some of the readers of the *Dental Practitioner and Advertiser*. Not that I employ different materials from any one else, or use different methods, but I certainly do accomplish the result without pain.

With occasional exceptions, I adjust the rubber dam where arsenic is to be applied, then bathe the cavity thoroughly with carbolic acid, and remove the decayed dentine as thoroughly as possible without producing unnecessary pain. I find it desirable, as others do, to have a large surface of exposure to which to apply the arsenic, but I apply it to a small exposure rather than give the patient pain in the attempt to enlarge it. I now place two or three drops of carbolic acid upon a glass slab, and add as much of the hydrochlorate of coçaine as it will dissolve. A pledget of cotton, supersaturated with this solution, is placed in the cavity and left there while I am preparing the paste. I have in a small bottle a preparation consisting of equal parts of acidum arsenicosum and morphinum muriaticum, with just enough carbolic acid to hold them together (not to make a paste). I take a bit of this, a little larger than a pinhead, and make a paste of it with the saturated solution of cocaine in carbolic acid. I now remove the pledget of cotton from the cavity, take the paste upon the point of a suitably shaped excavator, and apply it directly to the point of exposure. Over this I place a small, flat pledget of cotton, well saturated with the cocaine-carbolic acid solution, being careful not to let the cotton extend over the margin anywhere, and avoiding every trace of pressure.

As far as the action of the cocaine is concerned, I have no doubt the same result may be obtained by incorporating the crystals with the ordinary thin arsenic paste usually employed. So far as I know, the following formula, which has been repeatedly recommended, would serve the same purpose:

R— Acidi Arsenicosi,
Cocaine Hydrochlorate, aa, 0.5
Acidi Carbolici, q. s.
Ut fiat pasta mollis.

Personally, I have always used the fresh crystals of cocaine.

Now comes a very important part of the operation, that of retaining the application in position. If I had my enemy in the chair, and wished to make him atone in one night for all the sins he had ever committed, I would take some cotton, roll it up into a hard ball, saturate it with sandarac varnish, and force it into the cavity. The use of cotton and sandarac for retaining applications to the pulp appears to me to be utterly inexcusable. I think that one would be justified in calling it not only irrational, but slovenly practice. More or less pressure is absolutely necessary to make the cotton stay in place, and this is sure to increase the probability of pain in a high degree, to say nothing of the danger of causing minute quantities of the arsenic to exude and come into contact with the gums, while the cotton itself, unless packed very tight, soon becomes permeated with the secretions of the mouth.

The method of covering applications to the pulp with gutta-percha has also always appeared to me very objectionable, simply because it is next to impossible to cover the bottom of a cavity with a pledget of cotton, supersaturated as it should be with some local anæsthetic, and then fill over this cotton, wet with gutta-percha solution, so as to obtain anything like a watertight filling, without exerting pressure upon the cotton. For all cases where we have to enclose applications on cotton, the oxysulphate of zinc is vastly superior to gutta-percha. I use the preparation known as Fletcher's artificial dentine, but am not acquainted with preparations of a similar character which may be on the American market. I mix the preparation moderately thin, so that when it is taken upon the spatula it hangs down slightly. It should not, however, be thin enough to drop off. For inserting it, I use in most cases a very thin, sickle-shaped spatula. Taking a small quantity upon the end of the spatula, I draw it across the margin of the cavity, just about as one draws a plaster knife across the edge of a board to wipe the plaster off. I thereby fix the cotton on one margin; then in the same manner it is covered on the opposite margin, eventually a third or fourth portion being necessary to complete the operation. For approximal cavities in molars, an instrument bent upon its surface will sometimes be found preferable to a sickle-shaped one. The method of applying the cement is also somewhat different for molars, but a little experience will soon make the manner of manipulation apparent to every one. Like everything else it requires some practice.

With the oxy-phosphate, or even with plaster of Paris one can place a wet pledget of cotton in the open end of a tube ¼ inch in diameter, and fill over it without displacing the cotton, or exerting the least perceptible pressure upon it, a thing which cannot be done with gutta-percha, or any other material that I know of. My manner of applying arsenic was put to a severe test a few days ago in two quite similar cases, one of which I may relate. A middle-aged gentleman, of nervous temperament, presented himself, with an aching tooth on the right side of the upper jaw. An examination revealed a second molar decayed on the distal surface. The cavity contained a pledget of cotton, the removal of which was followed by a paroxysm of severe pain, and a drop of pus was seen to exude from the point of exposure of the pulp. I make it a rule never to apply arsenic to an aching or highly inflamed pulp, but in this case, for special reasons, I decided to deviate from the rule. I at once inserted a pledget of cotton, saturated with the cocaine-carbolic acid solution, which was allowed to remain about five minutes, the pain gradually diminishing in intensity; I then asked the patient to rinse his mouth (not a necessary part of the procedure, however,) and renewed the application, which I left in the cavity while I was preparing the arsenic paste. The latter was applied as described above. When I had finished, there was still some grumbling pain, which disappeared gradually and entirely inside of ten minutes, and the patient did not have a trace of pain afterwards. The pulp could be extirpated on the following day. Thus the application of arsenic was made the means of completely stilling the pain, instead of producing the violent suffering so often complained of. In another case, two days ago, a young lady came to me with an aching tooth which had troubled her more or less every day for weeks. It was so sensitive that the touch of the finger, unless quite warm, produced severe pain. I applied arsenic with my usual care, and from the moment she left my office she felt nothing whatever to remind her that she had a decayed tooth.

I attribute my success in this operation to the observance of the greatest possible delicacy in making the application to the pulp, in particular to the avoidance of every trace of pressure, and secondly to the maintenance of a constant anæsthetic condition by use of the cocaine-carbolic acid solution.—*Dental Pract. and Advertiser.*

